# Evidence for Divergence of the Genus ‘*Solwaraspora*’ Within the Bacterial Family *Micromonosporaceae*

**DOI:** 10.3390/microorganisms13071576

**Published:** 2025-07-04

**Authors:** Hailee I. Porter, Imraan Alas, Nyssa K. Krull, Doug R. Braun, Scott R. Rajski, Brian T. Murphy, Tim S. Bugni

**Affiliations:** 1 School of Pharmacy, University of Wisconsin-Madison, Madison, WI 53705, USA; haileeporter123@gmail.com (H.I.P.); imraanalas@gmail.com (I.A.); drbraun1@wisc.edu (D.R.B.); scott.rajski@wisc.edu (S.R.R.); 2 Laboratory of Genetics, University of Wisconsin-Madison, Madison, WI 53706, USA; 3 Department of Integrative Biology, University of Wisconsin-Madison, Madison, WI 53706, USA; 4 Department of Pharmaceutical Sciences, University of Illinois at Chicago, Chicago, IL 60607, USA; nkrull@uic.edu (N.K.K.); btmurphy@uic.edu (B.T.M.)

**Keywords:** biosynthetic gene cluster, drug discovery, genomics, marine-derived, natural products

## Abstract

The purpose of this study was to investigate the taxonomic and phylogenomic placement of the proposed genus ‘*Solwaraspora*’ within the context of other marine genera using a dual-omics approach. Initially, we isolated bacteria from marine tunicates, squirts, and sponges, which were morphologically similar to an emerging genus (identified as ‘*Micromonospora*_E’ by the GTDB-tk2 database using whole genome sequence data) by colony shape, size, and clustering pattern, but only found five strains in our dataset belonging to this distinction. Due to the minimally explored nature of this genus, we sought to identify more bacterial strains with similar morphology to *Micromonospora*‘*Micromonospora*_E’ by whole genome sequencing (WGS). Within our collection, we noted 35 strains that met this criterion and extracted genomic information to perform WGS on these strains. With this information, we studied taxonomic and phylogenomic relationships among these organisms. Using the data gathered from WGS, we were able to identify an additional five strains labeled by the GTDB-tk2 database as *Micromonospora*‘*Micromonospora*_E’, as well as construct phylogenomic trees to examine the evolutionary relationships between these strains. ANI values were calculated between strains from our dataset and type strains of *Micromonospora* and *Plantactinospora* as well as against an outgroup *Streptomyces* strain. No type strains are available for ‘*Solwaraspora*’. Using MALDI-TOF MS, we positively identified ‘*Solwaraspora*’, which was supported by the phylogenomic tree showing *Micromonospora*’*Micromonospora*_E’ (‘*Solwaraspora*’) in a distinct clade from *Plantactinospora* and *Micromonospora*. Additionally, we discovered gene cluster families (GCFs) in alignment with genera, as well as a large representation of biosynthetic gene clusters (BGCs) coming from the ‘*Solwaraspora*’ strains. These findings suggest significant potential to discover novel chemistry from ‘*Solwaraspora*’, adding to the importance of investigating this new genus of bacteria.

## 1. Introduction

Natural products (NPs) have been fundamental in their use for drug discovery and development, in particular for antimicrobials and anticancer drugs, but the drug discovery process has faced many challenges regarding optimization and prioritization of bacteria to investigate their drug potential [1]. Mostly, drug discovery efforts have focused on terrestrial microorganisms [2,3]. Notably, the actinobacterial genus *Streptomyces* has contributed heavily to the reservoir of identified NPs, especially for antibacterials [4,5]. Conversely, marine bacteria have not been explored to an appreciable extent. Marine microorganisms have become increasingly important in the field of drug discovery, as they have been shown to contain a wealth of promising NPs [6]. The family *Micromonosporaceae* has been well documented within the marine environment [7], including the genus *Salinospora*, comprising marine obligate bacteria [5]. We recently sequenced 38 genomes of bacteria isolated from the marine environment within the family *Micromonosporaceae* to evaluate their potential for drug discovery through analysis of biosynthetic gene clusters (BGCs) [6]. We found immense potential for novel natural product discovery. We also found that a new genus noted as *Micromonospora*‘*Micromonospora*_E’ by the Genome Taxonomy Database (GTDB) had BGCs that were more unique relative to *Micromonospora* spp., which supported this genus as a significant resource for drug discovery. To capitalize on the drug discovery potential of *Micromonospora*‘*Micromonospora*_E’, we attempted to expand our genomics studies to survey additional bacteria in our collection that could be *Micromonospora*‘*Micromonospora*_E’. We surveyed 16S rRNA gene sequences among strains that were possibly members of the *Micromonospora*‘*Micromonospora*_E’ (Figure S1). Previous work in our lab identified four novel species as *Micromonospora*‘*Micromonospora*_E’, representing a fourfold increase in the known *Micromonospora*‘*Micromonospora*_E’ strains when compared to the 311,480 bacterial strains in the GTDB-tk2 database [6].

Previously, Magarvey et al. proposed a new genus of *Micromonosporaceae*, designated as ‘*Solwaraspora*’, that was distinct enough from other genera within the family based on fatty acid profiles, respiratory menaquinones, and cell wall sugars differing from other genera of *Micromonosporaceae* [8]. Magarvey called for future work to show that ‘*Solwaraspora*’ can be classified in a distinct clade from other genera within the family *Micromonosporaceae*. We hypothesized that *Micromonospora*‘*Micromonospora*_E’ was in fact ‘*Solwaraspora*’, but this had not been proven. The current effort aimed to expand our genomics studies, confirm the ‘*Solwaraspora*’–*Micromonospora*’*Micromonospora*_E’ connection, and establish robust tools to identify ‘*Solwaraspora*’ using matrix-assisted laser desorption ionization time-of-flight mass spectrometry (MALDI-TOF MS) whole cell analysis in conjunction with the bioinformatics pipeline IDBac to provide a route to species-level identification. MALDI-TOF MS has been established as a technique for identifying bacteria via fingerprint mass spectra of known isolates [9]. IDBac uses protein MS fingerprints to distinguish taxa based on degree of overlap with NP spectra [10].

Sequencing techniques have been used to elucidate taxonomic information about many species, with 16S rRNA sequencing confirmed for bacterial species and strain level classification [11,12]. A major limitation to 16S rRNA sequencing is that there are often misclassified or unclassified results [12,13]. For example, when inputting 16S rRNA sequences from the 35 strains of bacteria in our dataset into NCBI, we observed strains of bacteria that were designated as *Micromonospora* when applying the RNA RefSeq search filter but were considered *Verrucosispora* when using the Core Nucleotide Database search (Figure S1, Table S1). This further illustrates the need for additional approaches for taxonomic classification within the bacterial family *Micromonosporaceae.* More recently, whole genome sequencing (WGS) paired with GTDB analysis has allowed for enhanced detection of microbial species with better accuracy [12,13]. However, the GTDB does not directly classify the genus *Verrucosispora* when directly used as input into the search function for strains in their database, though it does list the NCBI classification of the genus [14]. Average nucleotide identity (ANI) is the similarity between genomes that correspond to threshold values that can be applied to distinguish species. It is well documented that a 96.5% ANI value correlates to a 70% DNA-DNA hybridization (DDH) threshold for differentiating species using whole genomes [15]. For genera-level distinctions, the ANI values are found between 69.99 and 82.45 [15]. We used the platform FastANI to compute ANI values amongst strains in our dataset that evaluates a query draft genome by comparing it against the full collection of available prokaryotic genomes in the NCBI, including type strains [14]. For this study, we used a combination of complementary approaches to show divergence of ‘*Solwaraspora*’ from other genera within the family *Micromonosporaceae*.

Research showing the evolutionary divergence of ‘*Solwaraspora*’ within the bacterial family *Micromonosporaceae* is a current knowledge gap. Therefore, we propose and provide a rationale for the determination of the divergence of ‘*Solwaraspora*’ leveraging computational genomics and proteomics. Comparative genomic analysis through BGC clustering showed BGC similarity at the genus level but divergence at the family level, similar to what has been previously published [5]. At the whole genome level and BGC level, we saw species-level distinction even though the 16S rRNA sequences were largely identical. Therefore, we investigated MALDI-TOF MS in tandem with the IDBac pipeline to identify speciation of ‘*Solwaraspora*’, among other bacteria in the family *Micromonosporaceae*. We compared IDBac identification with genomics-based methods of identification to reinforce confidence in the results. This study shows the evolutionary divergence of ‘*Solwaraspora*’ from *Micromonospora* through (a) phylogenomic analysis, (b) comparative genomic analysis of secondary metabolites, and (c) protein-level expression.

## 2. Materials and Methods

### 2.1. Strain Isolation, Extraction, and Sequencing

Thirty-five strains of bacteria were chosen for sequencing based on colonial morphology to ‘*Solwaraspora*’ strains previously grown in the laboratory, and they were sequenced using PacBio sequel platforms using two Sequel single-molecule real-time (SMRT) cells [University of Wisconsin, Madison (UW-Madison), Biotechnology Center, Madison, WI]. These sequences were corrected, trimmed, and assembled with Canu v1.8 [16] with the parameter ‘genomeSize = 8m’ (UW-Madison, Center for High Throughput Computing, Madison, WI, USA). BUSCO v5.4.3 [17] and QUAST v5.0.2 [18] were used to verify genome assembly based on completeness and quality. Sequences were then aligned using multiple sequence alignment using MUSCLE v5 [19] and filtered for gaps in more than 10% of samples using trimAI v1.4 [20]. CompareM v0.1.2 [21] was used to produce average amino acid identity (AAI) amongst the 35 genomes with the GTDB (*n* = 35).

WGS fasta files were then run through Bioconda GToTree v1.8.8 [22] for construction, and the output .tre file was used as input for iTOL, Interactive Tree of Life v6.9.1 [23], for visualization of the tree using the parameters 0-degree rotation, position aligned, alignment left, rotation on, and midpoint rooted. GToTree uses estimates of genome completeness and redundancy to create a phylogenomic tree based on a specified single-copy gene (SCG) set, which was Actinobacteria for this study. Genome assemblies and ascension numbers are publicly available via the NCBI Genbank by strain name. ANI comparison values were computed using FastANI v1.34 [14] between each strain in the dataset and type strains of *Micromonospora echinospora* strain ATCC 15,837 and *Plantactinospora endophytica* NBRC 110,450, as well as an outgroup type strain of *Streptomyces clavuligenus* ATCC 27064. Type strains genomes were accessed from BacDive [24].

### 2.2. BGC Identification

antiSMASH v6.1.0 [25] was used with the detection strictness relaxed and, KnownClusterBlast, SubClusterBlast, and RREFinder checked to identify BGCs. BGCs with high similarity values and predicted product types with possible antibiotic activity were noted.

### 2.3. Product Class Classification and Analysis

Using the BiG-SCAPE tool [26], antiSMASH v6.1.0 GenBank files, pairwise distance across predicted BGCs, and sequence similarity networks were constructed with a default cutoff of 0.3 and parameters used by earlier work. The output of BiG-SCAPE was then imported to CytoScape v3.10.1 [27]. BGC product annotations generated by antiSMASH v6.1.0 were color coded based on BiG-SCAPE cluster classes [28] and taxonomy was categorized by node shape distinctions. This program uses ‘CORe Analysis of Syntenic Orthologues to prioritize Natural product gene clusters’ (CORASON), which provides phylogenetic relationships within and across bacterial families. MIBiG [29] BGC product types were identified and characterized with a different node shape. Singletons and doublets were removed for better visualization but still saved in the dataset and considered for future analysis.

### 2.4. MALDI-TOF MS Sample Preparation and IDBac Data Analysis

Samples from our laboratory were sent to the University of Illinois-Chicago where pure cultures were grown on A1 agar media for seven to nine days at ambient temperature. Following incubation, a 384-spot MALDI target plate (Bruker Daltonics, Billerica, MA, USA) was prepared by transferring a small volume of isolated colonies in five biological replicates, followed by a 1 μL overlay of 70% formic acid (7:3 Optima, Fisher Chemical: Optima LC-MS Grade Water Fisher Chemical) and α-cyano-4-hydroxycinnamic acid matrix (α-cyano-4-hydroxycinnamic acid powder, 98% pure, Sigma-Aldrich, part-C2020), 50% LC-MS grade acetonitrile, 47.5% LC-MS grade water, and 2.5% LC-MS grade trifluoroacetic acid). MALDI-TOF MS spectra were analyzed using IDBac version 1.1.10 (https://chasemc.github.io/IDBac/ (accessed on 16 December 2024)). The protein dendrogram was created using the following parameters: percent presence = 70; signal-to-noise ratio = 4; lower mass cutoff = 3000 Da; upper mass cutoff = 15,000 Da; ppm tolerance = 1000; distance algorithm = cosine; clustering algorithm = average (UPGMA), and presence/absence. The most up-to-date version of IDBac can be found at https://idbac.org/.

## 3. Results

### 3.1. Phylogenomic Analysis

**Figure 1:**
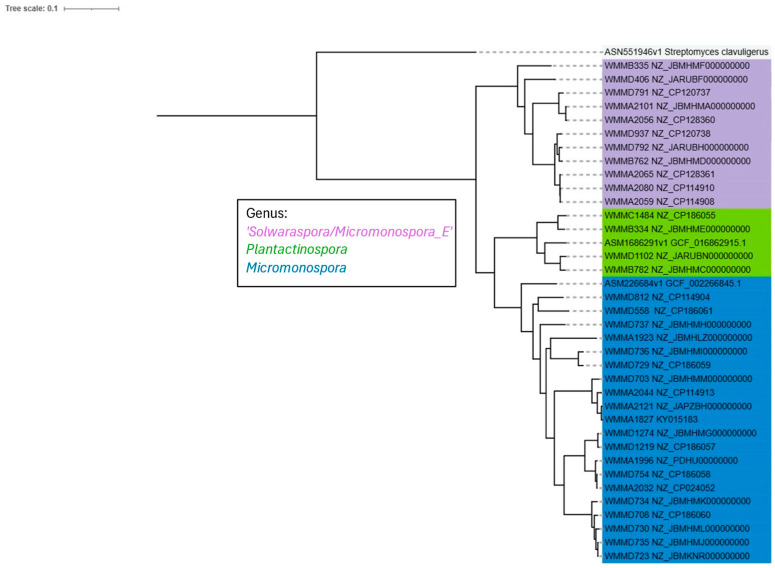
WGS maximum-likelihood phylogenomic tree for the strains of interest labeled by genus with a focus on ‘*Solwaraspora*’ and *Plantactinospora*. The tree was constructed using iTOL v6 with the output from GTo Tree. ‘*Solwaraspora*’ strains are in a separate clade from *Micromonospora* and *Plantactinospora* strains, with *Plantactinospora* more closely aligning with *Micromonospora* (n = 35). Type strain references were included for *Micromonospora* and *Plantactinospora*, as well as an outgroup type strain of *Streptomyces.* Strain name is accompanied by NCBI accession number. GenBank and RefSeq assemblies can be found via the supplemental materials. The tree scale represents bootstrap values calculated by MUSCLEv5 in GToTree.

WGS was performed on 35 bacterial strains and corresponding taxa were identified via the GTDB. GToTree was then utilized to construct a phylogenomic tree using orthologous SCG hits. This showed a clear divergence of the strains classified as ‘*Solwaraspora*’ from the strains classified as *Micromonospora*, both under the family *Micromonosporaceae* (Figure 1). Additionally, we observed that *Plantactinospora* was more closely related to *Micromonospora*, with strains of bacteria clading consistently with their genus classification. Strains in this dataset had not yet been classified. ANI values were computed and compared. WMMB335, a ‘*Solwaraspora*’ strain, had an ANI value of 79.5235 when compared to WMMD1219, a *Micromonospora* strain, and an ANI of 79.594 compared to WMMB334, a *Plantactinospora* strain. These are within the range of ANI values calculated for genera-level distinction [14]. The rest of the ANI values computed between each strain in a pairwise manner can be found in the Supplementary Materials. Type strains of *Micromonospora echinospora* and *Plantactinospora endophytica* were used in this analysis, accessed from BacDrive [24]. Type strain alignment with strains in this dataset by genera supports taxonomic classifications from the GTDB. An outgroup of *Streptomyces clavuligenus* further validates the phylogenomic relationships.

### 3.2. BGC Network Analysis

**Figure 2:**
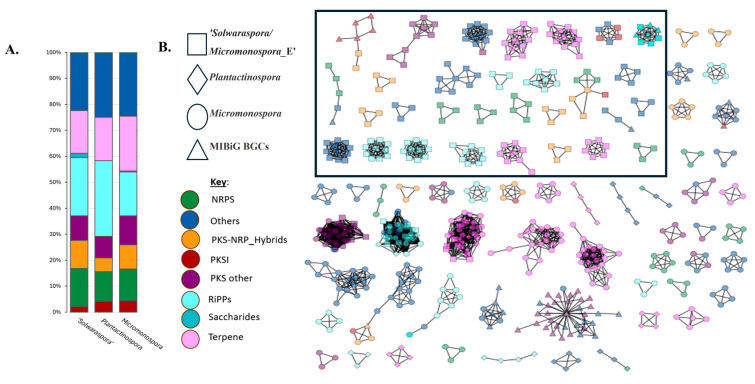
(**A**) Percentage of BGCs from the 35 strains by BiG-SCAPE predicted natural product BGC classification, including the singletons, and excluding MIBiG BGCs from the MIBiG repository. (**B**) CytoScape matrix visualization of BiG-SCAPE output for 35 total genomes’ BGC product type. Included in this visual are 657 total BGCs, with GCFs that contain 2 or fewer BGCs removed for better visualization, 168 of which were singletons and 41 were GCFs with 2 BGCs. The colors represent the different BGC product class as per BiG-SCAPE distinctions, and the node shape represents the taxonomy with emphasis on ‘*Solwaraspora*’ and *Plantactinospora*, and *Micromonospora*. There were 18 GCFs containing MIBiG BGCs. MIBiG repository BGCs are also represented by a differing node shape. A box is drawn around GCFs containing ‘*Solwaraspora*’ BGCs, as well as three GCFs that contained ‘*Solwaraspora*’ and MIBiG BGCs.

Next, we wanted to determine if there were genus-specific differences in BGC content, which would support categorizing ‘*Solwaraspora*’ as its own genus. Using WGS paired with the MIBiG database, we paired BGC content with taxonomic identity, further increasing the knowledge on marine microorganisms. To do so, we analyzed the same 35 strains for BGC content using antiSMASH v5.1.1 GenBank files as input for BiG-SCAPE with the goal of demonstrating the genera-specific BGCs. BiG-SCAPE calculates sequence similarity to form networks of BGCs, taking into account copy number, sequence identity, and protein domain content [26]. To visualize this data, we used Cytoscape [27] that utilizes BiG-SCAPE [26] program analysis of whole genome data. Each BGC is visualized as a node, with node shapes representing each genus in this dataset. In total, there were 784 BGCs in this dataset. We saw 58.03% of the BGCs coming from the 20 strains within the genus *Micromonospora,* 29.72% from the 11 strains of ‘*Solwaraspora*’, and 12.24% from the 4 *Plantactinospora* strains (Table S2). Of the 327 GCFs found in this dataset, there are 18 GCFs that have MIBiG repository BGCs, meaning that they have similarity to known BGCs. The other GCFs do not have MIBiG BGCs, suggesting that these are potentially novel BGCs (Figure 2B). GCFs comprising only BGCs from ‘*Solwaraspora*’ are highlighted, as well as GCFs containing only ‘*Solwaraspora*’ and MIBiG repository BGCs (Figure 2B). This shows 30 GCFs conserved within the genus ‘*Solwaraspora*’. From *Plantactinospora* and *Micromonospora,* we observed 9 and 36 GCFs, respectively. In addition, we noted distinct BGC clustering by genus. Within this dataset, there were 168 singleton BGCs.

### 3.3. MALDI-TOF MS/IDBac Analysis of ‘Solwaraspora’ *spp.*

**Figure 3:**
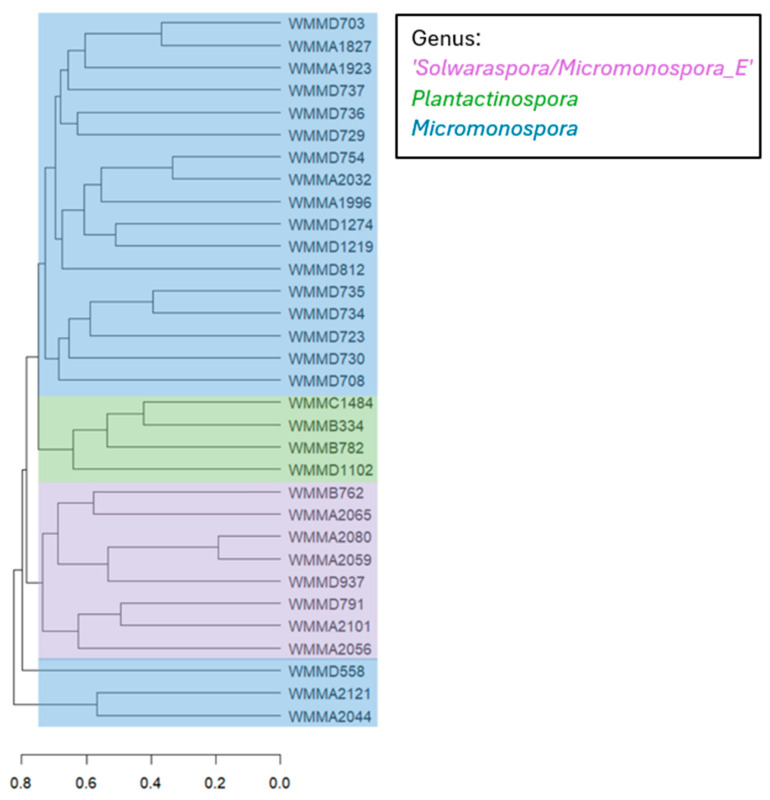
MALDI-TOF MS protein dendrogram constructed using IDBac. Strains WMMD406 and WMMA335 from the original dataset are absent from the dendrogram as there were difficulties with growth. The y axis relates individual samples, and the x axis shows the cosine score of the spectra vectors.

To demonstrate another way of verifying the genus ‘*Solwaraspora*’, we utilized an orthogonal approach via the IDBac desktop application, which uses MALDI-TOF MS protein data to organize bacteria through protein spectrum similarity and hierarchical clustering. A protein dendrogram of 8 ‘*Solwaraspora*’, 20 *Micromonospora* and 4 *Plantactinospora* strains was generated using cosine distance and average (UPGMA) hierarchical clustering (Figure 3). This approach allowed us to provide an additional method for classifying genera within the *Micromonosporaceae* family. From this, we noted distinct clustering of ‘*Solwaraspora*’ strains, consistent with findings from the phylogenomic WGS tree.

## 4. Discussion

### 4.1. WGS-Based Phylogenomic Evaluation of ‘Solwaraspora’ and Plantactinospora

Historically, the genus ‘*Solwaraspora*’ has been misidentified using only 16S rRNA sequences [23]. Using WGS-based taxonomic classification, we saw a higher fidelity than using only 16S rRNA sequencing. Bacterial strains often have identical sequences, suggesting identical species, but speciation can be distinguished when assessed at the whole genome level. Based on taxonomic assignments using WGS, we observed the emergence of the proposed genus ‘*Solwaraspora*’ from *Micromonospora* in higher fidelity compared to traditional 16S rRNA sequencing. Initially, we evaluated 28 strains of bacteria believed to belong to the family *Micromonosporaceae* and constructed a WGS phylogenomic tree, which produced a monophyletic clade of *Micromonospora* and a clade consisting of ‘*Solwaraspora*’ and *Plantactinospora* (Figure S1). To this preliminary dataset, we added seven strains previously identified in our lab as either ‘*Solwaraspora*’ or *Plantactinospora* by WGS. Following WGS phylogenomic tree construction with all 35 strains, we observed a clade containing 11 strains all designated ’*Micromonospora*_E’ by the GTDB, which we identified as ‘*Solwaraspora*’, followed by a clade consisting of 4 strains identified as *Plantactinospora* and 20 strains as varying species within the genera *Micromonospora* (Figure 1). In the larger clade, two distinct subclades helped differentiate *Plantactinospora* from *Micromonospora* (Figure 1). From this phylogenomic tree, we hypothesized that a common ancestor links all three genera but is further away in evolutionary distance from the common ancestor linking *Plantactinospora* and *Micromonospora*, therefore suggesting that ‘*Solwaraspora*’ is in fact its own genus, with a more distant genetic relatedness to both *Micromonospora* and *Plantactinospora* than the latter are to each other. Importantly, phylogenomic analyses provided the evidence necessary to designate ‘*Solwaraspora*’ as its own genus by ANI value threshold distinctions, which are highly validated for taxonomical classification [14]. This directly counters results obtained via 16S rRNA sequencing of the ‘*Solwaraspora*’ strains and provides a strong basis for the shift to viewing ‘*Solwaraspora*’ as its own genus.

### 4.2. BGC Analysis of Genera-Level Evolutionary Divergence

BGC analysis was performed using the same whole genome sequences to predict BGC content across the 35 strains of *Micromonosporaceae*. BiG-SCAPE, using the MIBiG repository for known BGCs, computes a distance matrix of relatedness between BGCs, which is visualized in Figure 2. Comparative genomic analysis has been shown to positively correlate BGC distance to evolutionary and phylogenomic differences [30]. BGC profiles in other microbial families were consistent with their phylogenetic relationships [30,31], which is the lens by which we assessed the BGC matrix. By doing so, we observed GCFs with genus-specific clustering, indicating that on the biosynthetic level there were also secondary metabolic patterns that distinguished ‘*Solwaraspora*’ from *Micromonospora* and *Plantactinospora*. As shown in Figure 2, we found 30 GCFs for ‘*Solwaraspora*’ BGCs that did not cluster with *Plantactinospora* or *Micromonospora*. On the BGC content level, we saw genus level differences in the abundance of BGC product types between genera as well as scaled BGC content by number of strains per genera in this dataset (Figure 2B). In total, 784 BGCs were identified, 68 of which were singletons, indicating they were either highly dissimilar to MIBiG BGCs that they did not cluster or represent novel BGCs not yet characterized (Figure 2A). The differences in BGC content between these three genera supported the idea of searching for new NPs from understudied bacteria. The BGCs present in the *Plantactinospora* and ‘*Solwaraspora*’ strains had limited similarity to those in MIBiG, suggesting that they may make novel NPs. In total, 12.24% of the BGCs belong to the four strains classified as *Plantactinospora*. Ten strains were classified as ‘*Solwaraspora*’ and accounted for 29.72% of the BGCs. The other 19 strains were classified within the genus *Micromonospora* and are represented by 58.035% of the BGCs. The BiG-SCAPE classification was based on the product annotation of antiSMASH that was separated into eight classes: PKS I, PKS other, NRPs, RiPPs, Saccharides, Terpene, PKS/NRPS hybrid, and others [31]. Out of these, the BiG-SCAPE classification of Saccharides accounted for the lowest percentage of BGC products across genera, RiPPs being the most abundant BGC product for ‘*Solwaraspora*’ and *Plantactinospora*, and ‘others’ being the most abundant BGC product for *Micromonospora* (Figure 2B).

### 4.3. IDBac Classification of Micromonospora, Plantactinospora, and ‘Solwaraspora’ by Protein MS Signatures

Because 16S rRNA sequencing was not sufficient for characterization of ‘*Solwaraspora*’ at the species level and ‘*Solwaraspora*’ can be difficult to distinguish based on gross morphology, we aimed to establish a robust method of identification. IDBac is a MALDI-MS bioinformatics platform used to group bacteria based on the similarity of MS protein profiles [10]. By leveraging IDBac in parallel with WGS-based taxonomy, we were able to identify strains as ‘*Solwaraspora*’ via protein analysis (Figure 3). For a more thorough understanding of the mode of evolutionary divergence, we are additionally conducting further studies to investigate ribosomal protein sequences as a source of genetic diversity in these strains of bacteria. This may provide a mechanism for explaining differences seen in phylogenomic trees made with whole genome sequence similarity versus dendrograms made with MALDI-TOF MS when comparing marine bacterial strains. The importance of this is to provide rationale for further investigation into strains that have highly similar 16S rRNA sequences but may differ significantly on the level of secondary metabolites.

Importantly, we observed clustering of ‘*Solwaraspora*’ in the IDBac dendrogram (Figure 3), suggesting a possible mode of divergence for ‘*Solwaraspora*’, and a way to potentially identify candidate biomarkers markers within similarity groupings, using IDBac, though further studies are needed to confirm this function of the platform.

## 5. Conclusions

Using genomics, metabolomics, and MS-based proteomics, we support the distinction of the genus ‘*Solwaraspora*’ within the family *Micromonosporaceae*, with the goal of improving the taxonomical classifications of marine bacteria for natural product discovery prioritization. Notably, we demonstrate the potential of WGS-based taxonomy with MS-based proteomics to resolve differences in phylogenomic classification that were otherwise not possible using 16S rRNA analysis alone. Phylogenomic analysis of WGS data yielded a distinct clade of bacteria identified as ‘*Solwaraspora*’. Through MIBiG network analysis, BGC trends showed GCF association by genus, with many GCFs being specific to ‘*Solwaraspora*’ BGCs. This suggested that genera of bacteria in the family *Micromonosporaceae* demonstrated genus-level differences in secondary metabolite production, consistent with previous studies [5]. It should be noted that, without formal taxonomic criteria, genomic data remains provisional. Utilizing IDBac, we observed positive identification of strains of ‘*Solwaraspora*’ via their protein signature, again confirming the distinction between ‘*Solwaraspora*’, *Micromonospora*, and *Plantactinospora*. WGS, BGC analysis, and taxonomic classification by IDBac provide sufficient tools for analyzing taxonomic evaluations in marine bacteria and highlight the substantial natural product potential of the bacteria within the family *Micromonosporaceae.*

## Data Availability

The original contributions presented in this study are included in the article/supplementary material. Further inquiries can be directed to the corresponding author.

## Supplementary Materials

The following supporting information can be downloaded at: https://www.mdpi.com/article/10.3390/microorganisms13071576/s1. fastANI ANI Values, fastANI Type Strains, Genome Ascension Numbers, GTDB Summary, GToTree Summary, Supplementary Materials: Figure S1. 16S rRNA Phylogenomic Tree; Figure S2. Phylogenomic Tree of 28 Genomes; Figure S3. Images of Plates; Table S1. GTDB and NCBI Comparison; Table S2. Genome Accession Numbers; Table S3. GToTree Summary; Table S4. AntiSMASH Predictions.
