# Supplementary material for: Evidence for Divergence of the Genus ‘Solwaraspora’ Within the Bacterial Family Micromonosporaceae
Source: Microorganisms. 2025 Jul 4;13(7):1576. doi: 10.3390/microorganisms13071576 (PMC12299039; doi:10.3390/microorganisms13071576)

Hailee I. Porter[1], Imraan Alas[1], Nyssa K. Krull[2], Doug R. Braun[1], Brian T. Murphy[2], Tim S. Bugni[1*]

[1]*School of Pharmacy, University of Wisconsin-Madison, Madison, Wisconsin, USA*

[2]*Department of Pharmaceutical Sciences, University of Illinois at Chicago, Chicago, Illinois, USA*

Corresponding Author: Tim Bugni, tim.bugni@wisc.edu

Table of Contents

Tables

Table S1. GTDB and NCBI Comparison

Table S2. Genome Accession Numbers

Table S3. GToTree Summary

Table S4. AntiSMASH Predictions

Figures

Figure S1. 16S rRNA Phylogenetic Tree

Figure S2. Phylogenetic Tree of 28 Genomes

Figure S3. Images of Plates

Table S1. GTDB and NCBI Comparison. All *Verrucosispora* submissions in GTDB. Shows NCBI taxonomy and GTDB taxonomy. Despite NCBI taxonomy for highlighted strains being named *Verrucosispora*, the actual NCBI taxonomy does not include *Verrucosispora* in the listed taxonomy (see column 3).

accession	ncbi_organism_name	ncbi_taxonomy	gtdb_taxonomy
GCA_018912195.1	Verrucosispora sioxanthis CWR15_Gen1	d__Bacteria; p__Actinomycetota; c__Actinomycetes; o__Micromonosporales; f__Micromonosporaceae; g__Micromonospora; s__Verrucosispora sioxanthis	d__Bacteria; p__Actinomycetota; c__Actinomycetes; o__Mycobacteriales; f__Micromonosporaceae; g__Micromonospora; s__Micromonospora sioxanthis
GCF_002879555.1	Verrucosispora sp. ts21	d__Bacteria; p__Actinomycetota; c__Actinomycetes; o__Micromonosporales; f__Micromonosporaceae; g__Micromonospora; s__	d__Bacteria; p__Actinomycetota; c__Actinomycetes; o__Mycobacteriales; f__Micromonosporaceae; g__Micromonospora; s__Micromonospora gifhornensis
GCF_003977705.1	Verrucosispora sp. FIM060022	d__Bacteria; p__Actinomycetota; c__Actinomycetes; o__Micromonosporales; f__Micromonosporaceae; g__Micromonospora; s__	d__Bacteria; p__Actinomycetota; c__Actinomycetes; o__Mycobacteriales; f__Micromonosporaceae; g__Micromonospora; s__Micromonospora gifhornensis
GCF_004307965.1	Verrucosispora sp. SN26_14.1	d__Bacteria; p__Actinomycetota; c__Actinomycetes; o__Micromonosporales; f__Micromonosporaceae; g__Micromonospora; s__	d__Bacteria; p__Actinomycetota; c__Actinomycetes; o__Mycobacteriales; f__Micromonosporaceae; g__Micromonospora; s__Micromonospora sp004307965
GCF_010560025.1	Verrucosispora sioxanthis	d__Bacteria; p__Actinomycetota; c__Actinomycetes; o__Micromonosporales; f__Micromonosporaceae; g__Micromonospora; s__Verrucosispora sioxanthis	d__Bacteria; p__Actinomycetota; c__Actinomycetes; o__Mycobacteriales; f__Micromonosporaceae; g__Micromonospora; s__Micromonospora sioxanthis
GCF_013364215.1	Verrucosispora sp. NA02020	d__Bacteria; p__Actinomycetota; c__Actinomycetes; o__Micromonosporales; f__Micromonosporaceae; g__Micromonospora; s__	d__Bacteria; p__Actinomycetota; c__Actinomycetes; o__Mycobacteriales; f__Micromonosporaceae; g__Micromonospora; s__Micromonospora sp004307965
GCF_0189122	Verrucosispora	d__Bacteria;	d__Bacteria; p__Actinomycetota;

05.1	sioxanthis CWR15_Gen3	p__Actinomycetota; c__Actinomycetes; o__Micromonosporales; f__Micromonosporaceae; g__Micromonospora; s__Verrucosispora sioxanthis	c__Actinomycetes; o__Mycobacteriales; f__Micromonosporaceae; g__Micromonospora; s__Micromonospora sioxanthis
GCF_027460185.1	Verrucosispora sp. WMMA2121	d__Bacteria; p__Actinomycetota; c__Actinomycetes; o__Micromonosporales; f__Micromonosporaceae; g__Micromonospora; s__	d__Bacteria; p__Actinomycetota; c__Actinomycetes; o__Mycobacteriales; f__Micromonosporaceae; g__Micromonospora; s__Micromonospora sioxanthis
GCF_027497155.1	Verrucosispora sp. WMMA2044	d__Bacteria; p__Actinomycetota; c__Actinomycetes; o__Micromonosporales; f__Micromonosporaceae; g__Micromonospora; s__	d__Bacteria; p__Actinomycetota; c__Actinomycetes; o__Mycobacteriales; f__Micromonosporaceae; g__Micromonospora; s__Micromonospora sioxanthis
GCF_027497175.1	Verrucosispora sp. WMMD573	d__Bacteria; p__Actinomycetota; c__Actinomycetes; o__Micromonosporales; f__Micromonosporaceae; g__Micromonospora; s__	d__Bacteria; p__Actinomycetota; c__Actinomycetes; o__Mycobacteriales; f__Micromonosporaceae; g__Micromonospora; s__Micromonospora sp027497175
GCF_027497295.1	Verrucosispora sp. WMMC514	d__Bacteria; p__Actinomycetota; c__Actinomycetes; o__Micromonosporales; f__Micromonosporaceae; g__Micromonospora; s__	d__Bacteria; p__Actinomycetota; c__Actinomycetes; o__Mycobacteriales; f__Micromonosporaceae; g__Micromonospora; s__Micromonospora sioxanthis
GCF_029581255.1	Verrucosispora sp. WMMD1129	d__Bacteria; p__Actinomycetota; c__Actinomycetes; o__Micromonosporales; f__Micromonosporaceae; g__Micromonospora; s__	d__Bacteria; p__Actinomycetota; c__Actinomycetes; o__Mycobacteriales; f__Micromonosporaceae; g__Micromonospora; s__Micromonospora sediminimaris

Table S2. Genome Accession Numbers

Strain	GenBank	RefSeq	NCBI
WMMA1827			
WMMA1923	GCA_049179195.1	GCF_049179195.1	NZ_JBMHLZ000000000
WMMA1996	GCA_002573675.1	GCF_002573675.1	NZ_PDHU00000000
WMMA2032	GCA_027497155.1	GCF_027497155.1	NZ_CP024052
WMMA2044	GCA_002688545.1	GCF_002688545.1	NZ_CP114913
WMMA2056	GCA_030345095.1	GCF_030345095.1	NZ_CP128360
WMMA2059	GCA_027497315.1	GCF_027497315.1	NZ_CP114908
WMMA2065	GCA_030345075.1	GCF_030345075.1	NZ_CP128361
WMMA2080	GCA_027497395.1	GCF_027497395.1	NZ_CP114910
WMMA2101	GCA_049179175.1	GCF_049179175.1	NZ_JBMHMA000000000
WMMA2121	GCA_027460185.1	GCF_027460185.1	NZ_JAPZBH000000000
WMMB334	GCA_049179095.1	GCF_049179095.1	NZ_JBMHME000000000
WMMB335	GCA_049179075.1	GCF_049179075.1	NZ_JBMHMF000000000
WMMB762	GCA_049179115.1	GCF_049179115.1	NZ_JBMHMD000000000
WMMB782	GCA_049179135.1	GCF_049179135.1	NZ_JBMHMC000000000
WMMC1484	GCA_049315195.1	GCF_049315195.1	NZ_CP186055
WMMD1102	GCA_029626265.1	GCF_029626265.1	NZ_JARUBN000000000
WMMD1219	GCA_049315215.1	GCF_049315215.1	NZ_CP186057
WMMD1274	GCA_049179035.1	GCF_049179035.1	NZ_JBMHMG000000000
WMMD406	GCA_029626025.1	GCF_029626025.1	NZ_JARUBF000000000
WMMD558	GCA_049315255.1	GCF_049315255.1	NZ_CP186061
WMMD703	GCA_049178935.1	GCF_049178935.1	NZ_JBMHMM000000000
WMMD708	GCA_049315245.1	GCF_049315245.1	NZ_CP186060
WMMD723	GCA_049863385.1	GCF_049863385.1	NZ_JBMKNR000000000
WMMD729	GCA_049315225.1	GCF_049315225.1	NZ_CP186059
WMMD730	GCA_049178955.1	GCF_049178955.1	NZ_JBMHML000000000
WMMD734	GCA_049178975.1	GCF_049178975.1	NZ_JBMHMK000000000
WMMD735	GCA_049178995.1	GCF_049178995.1	NZ_JBMHMJ000000000
WMMD736	GCA_049179015.1	GCF_049179015.1	NZ_JBMHMI000000000
WMMD737	GCA_049179055.1	GCF_049179055.1	NZ_JBMHMH000000000
WMMD754	GCA_049315235.1	GCF_049315235.1	NZ_CP186058
WMMD791	GCA_029581195.1	GCF_029581195.1	NZ_CP120737

WMMD792	GCA_029626105.1	GCF_029626105.1	NZ_JARUBH000000000
WMMD812	GCA_027497215.1	GCF_027497215.1	NZ_CP114904
WMMD937	GCA_029581175.1	GCF_029581175.1	NZ_CP120738

Table S3. GToTree Summary. Summary output from GToTree phylogenetic tree construction

Assembly ID	Taxonomy ID	# SCG Hits	Unique SCG Hits	% Comp	% Redundancy	# Hits after Filter	In final tree?
WMMA1827	NA	136	119	98.55	16.67	117	Yes
WMMA1923	NA	136	130	98.55	4.35	127	Yes
WMMA1996	NA	137	127	99.28	7.97	124	Yes
WMMA2032	NA	137	129	99.28	6.52	125	Yes
WMMA2044	NA	136	128	98.55	5.8	126	Yes
WMMA2056	NA	135	128	97.83	5.07	125	Yes
WMMA2059	NA	135	132	97.83	2.17	124	Yes
WMMA2065	NA	135	130	97.83	3.62	122	Yes
WMMA2080	NA	136	132	98.55	2.9	124	Yes
WMMA2101	NA	136	129	98.55	5.07	126	Yes
WMMA2121	NA	136	121	98.55	14.49	119	Yes
WMMB334	NA	136	130	98.55	5.07	127	Yes
WMMB335	NA	136	129	98.55	7.25	123	Yes
WMMB762	NA	137	130	99.28	5.07	122	Yes
WMMB782	NA	136	127	98.55	9.42	123	Yes
WMMC1484	NA	136	133	98.55	2.9	129	Yes
WMMD1102	NA	137	128	99.28	6.52	121	Yes
WMMD1219	NA	136	129	98.55	5.8	125	Yes
WMMD1274	NA	136	127	98.55	7.97	123	Yes
WMMD406	NA	136	130	98.55	5.07	125	Yes

WMMD558	NA	137	133	99.28	2.9	129	Yes
WMMD703	NA	136	129	98.55	5.07	127	Yes
WMMD708	NA	136	129	98.55	5.07	127	Yes
WMMD723	NA	136	129	98.55	5.07	125	Yes
WMMD729	NA	136	129	98.55	5.07	127	Yes
WMMD730	NA	136	129	98.55	6.52	127	Yes
WMMD734	NA	136	129	98.55	5.8	128	Yes
WMMD735	NA	136	127	98.55	7.25	122	Yes
WMMD736	NA	137	116	99.28	15.22	113	Yes
WMMD737	NA	137	124	99.28	11.59	121	Yes
WMMD754	NA	137	129	99.28	7.25	126	Yes
WMMD791	NA	136	127	98.55	7.25	124	Yes
WMMD812	NA	134	128	97.1	4.35	127	Yes
WMMD937	NA	136	129	98.55	5.07	122	Yes

Table S4. AntiSMASH Predictions. AntiSMASH above or equal to 60% similar known cluster predictions.

Strain	*Genus*	BGCs with >60% Similarity
WMMD937	*Solwaraspora*	matlystatin A (&2%), Alkyl-O-dihydrogeranyl-methoxyhydroquinones (71%), desferroxiamine (75%), tylactone (65%), macrotermycin (61%)
WMMD791	*Solwaraspora*	desferroxiamine E (75%),Alkyl-O-dihydrogeranyl-methoxyhydroquinones (71%)
WMMD406	*Solwaraspora*	coelibactin (90%), desferroziamine B/E (66%), hatomarubigin A-D (75%), Alkyl-O-dihydrogeranyl-methoxyhydroquinones (71%)
WMMB762	*Solwaraspora*	desferroxiamine E (75%), matylystatin A (66%), Alkyl-O-dihydrogeranyl-methoxyhydroquinones (71%)
WMMB335	*Solwaraspora*	salinipostin G (100%), SapB (75%), Alkyl-O-dihydrogeranyl-methoxyhydroquinones (95%), A33853 (60%), cystargolide A/B (60%)
WMMA2101	*Solwaraspora*	Alkyl-O-dihydrogeranyl-methoxyhydroquinones (71%), vancosamine (100%), desferroxiamine E (75%)
WMMA2080	*Solwaraspora*	desferroxiamine E (75%), macrotermycins (61%), Alkyl-O-dihydrogeranyl-methoxyhydroquinones (71%)
WMMA2065	*Solwaraspora*	coelibactin (90%), desferroxiamine E (75%), Alkyl-O-dihydrogeranyl-methoxyhydroquinones (71%), macrotermycins (61%)
WMMA2059	*Solwaraspora*	macrotermycins (61%), desferroxiamine E (75%), Alkyl-O-dihydrogeranyl-methoxyhydroquinones (71%)
WMMA2056	*Solwaraspora*	cyclomarin D (95%), Alkyl-O-dihydrogeranyl-methoxyhydroquinones (71%), vancosamine (100%), desferroxiamine E (75%)
WMMD1102	*Plantactinospora*	catenulipeptin (60%), erythrochelin (85%), Alkyl-O-dihydrogeranyl-methoxyhydroquinones (85%)
WMMB782	*Plantactinospora*	Alkyl-O-dihydrogeranyl-methoxyhydroquinones (85%), erythrochelin (85%), catenulipeptin (60%)
WMMC1484	*Plantactinospora*	Alkyl-O-dihydrogeranyl-methoxyhydroquinones (85%0, erythrochelin (71%), SapB (75%)
WMMB334	*Plantactinospora*	catenulipeptin (60%), Alkyl-O-dihydrogeranyl-methoxyhydroquinones (85%)
WMMA1923	*Micromonospora*	SapB (75%), Alkyl-O-dihydrogeranyl-methoxyhydroquinones (85%), desferroxiamine E (100%)
WMMD737	*Micromonospora*	SapB (80%), Alkyl-O-dihydrogeranyl-methoxyhydroquinones (71%), alkyl resorcinol (100%)
WMMD812	*Micromonospora*	desferroxiamine B (100%), Alkyl-O-dihydrogeranyl-methoxyhydroquinones (80%)
WMMD558	*Micromonospora*	Alkyl-O-dihydrogeranyl-methoxyhydroquinones (85%), desferroxiamine (100%), SapB (100%), WS79089A/hexaricin B/C (63%)
WMMD736	*Micromonospora*	desferroxiamine B (100%), SapB (75%), Alkyl-O-dihydrogeranyl-methoxyhydroquinones (71%)
WMMD729	*Micromonospora*	desferroxiamine B (100%), SapB (100%), Alkyl-O-dihydrogeranyl-methoxyhydroquinones (71%)
WMMD703	*Micromonospora*	calicreamicin (71%), SapB (75%), Alkyl-O-dihydrogeranyl-methoxyhydroquinones (85%)
WMMA2044	*Micromonospora*	desferroxiamine (83%), SapB (75%), Alkyl-O-dihydrogeranyl-

		methoxyhydroquinones (85%)
WMMA2121	*Micromonospora*	SapB (100%), desferroxiamine (83%)
WMMA1827	*Micromonospora*	aAlkyl-O-dihydrogeranyl-methoxyhydroquinones (71%), desferroxiamine (83%), SapB (100%)
WMMD1274	*Micromonospora*	SapB (75%), Alkyl-O-dihydrogeranyl-methoxyhydroquinones (71%), arenimycin A (85%), desferroxiamine E (100%)
WMMD1219	*Micromonospora*	Alkyl-O-dihydrogeranyl-methoxyhydroquinones (71%), SapB (75%), desferroxiamine E (100%)
WMMD1996	*Micromonospora*	desferroxiamine E (100%), actinorhodin (72%)
WMMD754	*Micromonospora*	Alkyl-O-dihydrogeranyl-methoxyhydroquinones (71%), actinorhodin (72%),desferroxiamine E (100%)
WMMD2032	*Micromonospora*	desferroxiamine E (100%), actinorhodin (72%), Alkyl-O-dihydrogeranyl-methoxyhydroquinones (72%)
WMMD734	*Micromonospora*	olimycin A/B (70%), SapB (75%), desferroxiamine E (100%), lobosamide A-C (86%), Alkyl-O-dihydrogeranyl-methoxyhydroquinones (71%)
WMMD708	*Micromonospora*	Alkyl-O-dihydrogeranyl-methoxyhydroquinones (71%), SapB (75%), desferroxiamine E (100%), lobosamide A-C (86%)
WMMD730	*Micromonospora*	Alkyl-O-dihydrogeranyl-methoxyhydroquinones (71%), SapB (75%), desferroxiamine E (100%), lobosamide A-C (84%), chuangxihmycin (60%)
WMMD735	*Micromonospora*	Alkyl-O-dihydrogeranyl-methoxyhydroquinones (71%), SapB (75%), desferroxiamine E (100%),chuangxihmycin (60%) pyralomicin 1a (74%), thiazostatin (86%)
WMMD723	*Micromonospora*	Alkyl-O-dihydrogeranyl-methoxyhydroquinones (71%), chuangxihmycin (60%), pyramicin 1a (74%), lobosamide (A-C) (84%), desferroxiamine E (100%), SapB (100%), thiazostatin (86%)

Figure S1. 16S rRNA Phylogenetic Tree. 16S rRNA sequence phylogenetic tree with NCBI taxonomical genus classifications. Constructed using Trex

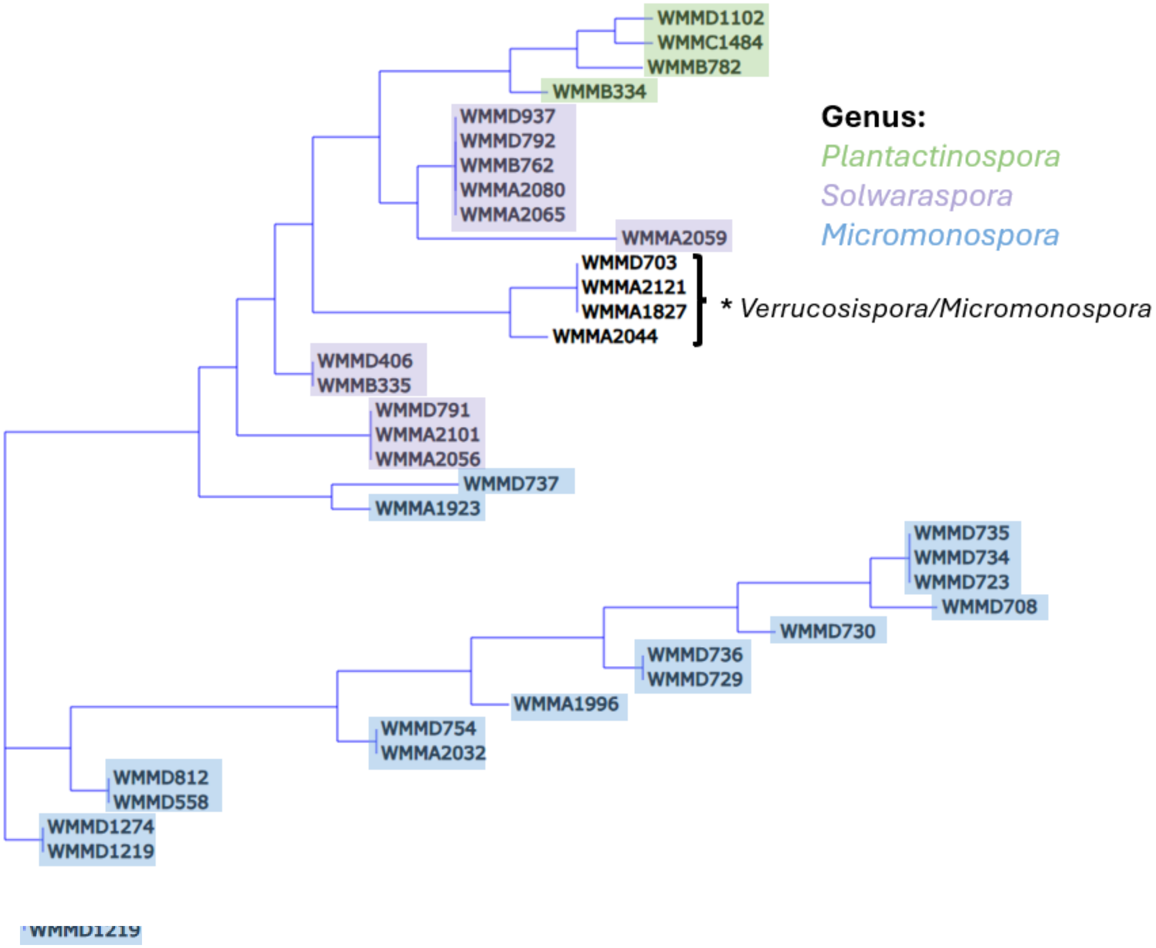

Figure S2. Phylogenetic Tree of 28 Genomes. Preliminary WGS phylogenetic tree with 28 genomes. *Solwaraspora* and *Plantactinospora* cluster together in one clade, with *Micromonospora* forming a monophyletic clade.

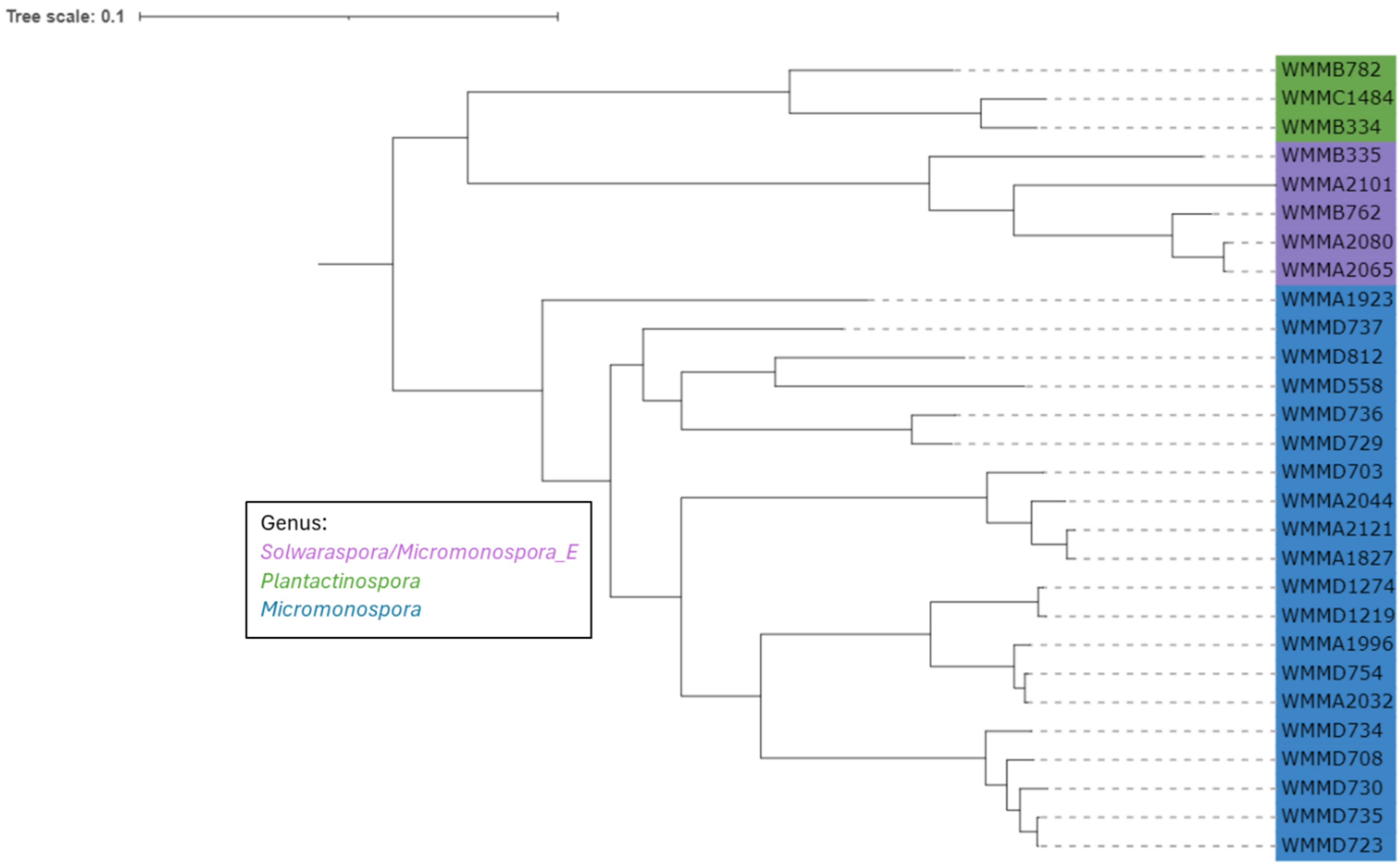

Figure S3 Images of *Micromonosporaceae*. Examples of *Micromonosporaceae* morphology used as visual prioritization for strain selection.

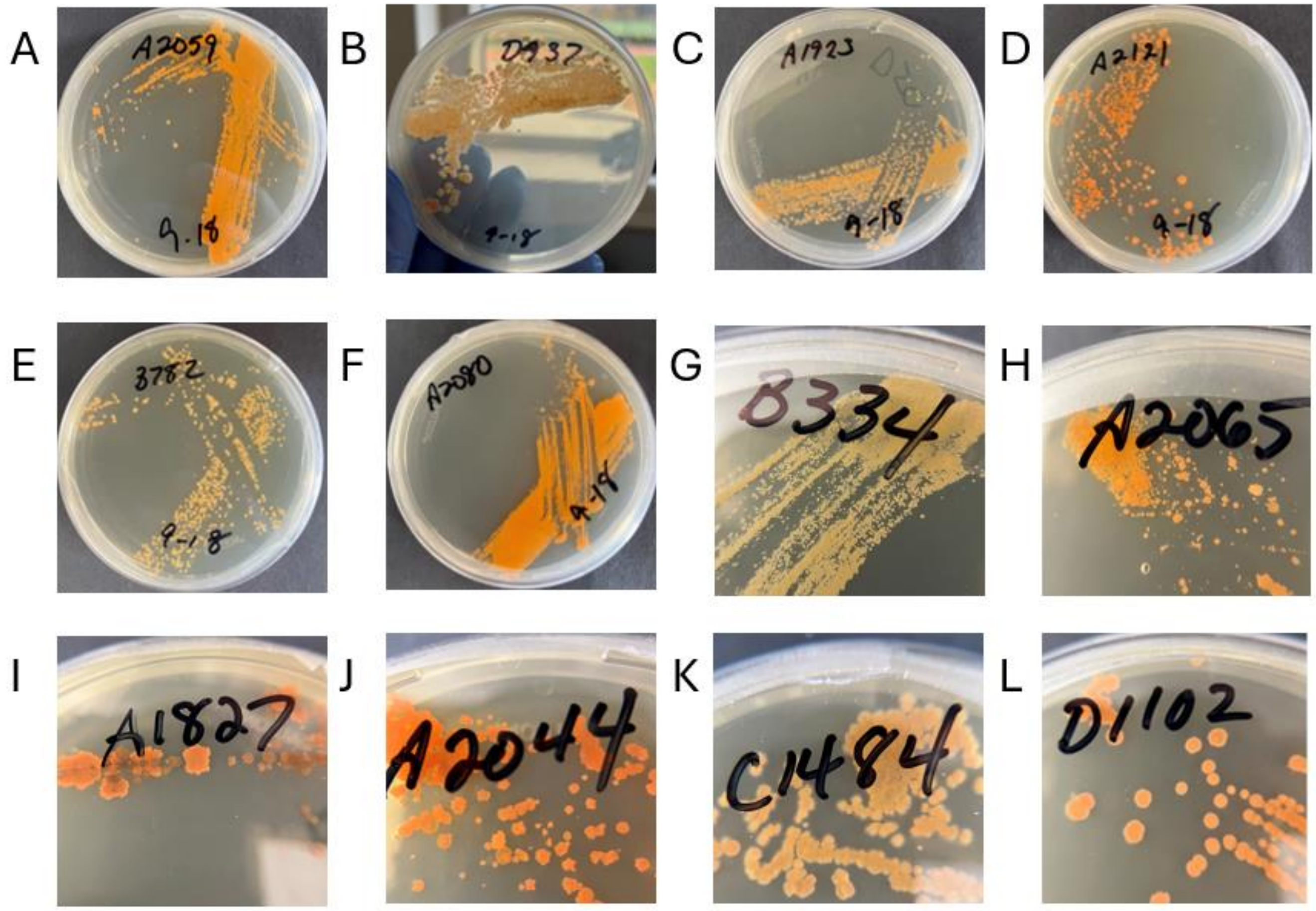

Supplement: Supplementary file 1 [file microorganisms-13-01576-s001.zip › Supplementary Materials (1).pdf]
